# Chromium (VI) – induced stress response in the plant *Plantago ovata* Forsk in vitro

**DOI:** 10.1186/s41021-018-0109-0

**Published:** 2018-10-15

**Authors:** Debangana Kundu, Sankalan Dey, Sarmistha Sen Raychaudhuri

**Affiliations:** Department of Biophysics, Molecular Biology, and Bioinformatics, University of Calcutta, 92, APC Road, Kolkata, 700009 India

**Keywords:** *Plantago ovata*, Hexavalent chromium (Cr VI), Stress, Polyphenols, Polyphenol oxidase (PPO), Phenylalanine ammonia lyase (PAL), Tolerance

## Abstract

**Background:**

Plants experience severe physiological stress from heavy metal pollution caused by improper discarding of the industrial wastes. Hexavalent chromium [Cr (VI)] is one of the major heavy metal pollutants in India and is present particularly in some regions where *Plantago ovata* grows to a great extent. This study was aimed at finding the effects of Cr (VI) on *P. ovata* and manoeuvres of the plant to combat such heavy metal exposure in vitro.

**Methods:**

Potassium dichromate was used as a source of Cr (VI) to induce the heavy metal stress. Range of Cr (VI) sublethal doses [0 mM (control), 0.1 mM, 0.3 mM, 0.5 mM, 1 mM, 1.5 mM and1.8 mM] was used to observe its effect on the plant. The seeds of the plant were grown on sucrose-agar media with different concentrations of potassium dichromate, and ten-day old seedlings were then harvested and examined.

**Results:**

The germination rate reduced below 50% at 1.9 mM Cr (VI) concentration and thus, 0 mM–1.8 mM concentration ranges were found to be suitable for sublethal dose. Morphological changes namely, reduction of the shoot-root length and multiple root development were caused by Cr (VI) in a dose-dependent manner. The plant showed elevated responses against Cr (VI), up to 1.5 mM (10 days treated) in terms of increasing accumulation of secondary metabolites like polyphenols, chlorophyll content (chlorophyll a, b and total chlorophyll), carotenoids and total antioxidant activity. DPPH radical scavenging activity along with malondialdehyde (MDA) content was not significantly elevated with the increase in Cr (VI) concentration indicating that the lipid peroxidation rate within the tissue was low. Phenylalanine ammonia lyase (*PAL*) and polyphenol oxidase (*PPO*) gene expressions were upregulated by 1 mM Cr (VI) concentration, which decreased at higher concentrations. The atomic absorption spectroscopy analysis also showed significant accumulation of Cr (VI) in the shoot and root with an increase in the potassium dichromate concentration.

**Conclusion:**

Cr (VI) reduced the shoot-root length and seed germination in a dose-dependent manner. The plant system tried to combat the Cr (VI) stress by upregulating the stress response genes in the phenylpropanoid pathway along with an increase in polyphenol and antioxidant contents, which were evident from the lowering of lipid peroxidation rate and increase in *PAL* and *PPO* gene expressions.

**Electronic supplementary material:**

The online version of this article (10.1186/s41021-018-0109-0) contains supplementary material, which is available to authorized users.

## Background

Heavy metals are the natural constituents of soil, but can cause pollution due to unregulated disposal which increases their concentration in soil and induces stress to both plants and animals. Plants growing on such contaminated soil were found to show several physiological changes to combat the heavy metal stress. Chromite is a natural form of chromium (Cr) and is found in ultramafic and serpentine rocks in various forms such as, crocoite (PbCrO_4_), tarapacite (K_2_CrO_4_), etc. [1]. Chromium is also one of the heavy metal pollutants, produced from various sources like steel industries, metal smelters, leather tanning, emissions from industries and also, from pesticide and fertilizer use [2–5]. Reported documents on hexavalent chromium [Cr (VI)] pollution estimated at various parts of the world, revealed its presence in the different industrial regions of India. Cr pollutants are found to be present in the industrial zone of Vadodara [6]. The soil of Surat, Gujarat also contains various heavy metal pollutants of which Cr was reported to be 305.2 mg/kg of soil which is higher than the permissible limit prescribed by the WHO guidelines [7].

Cr is found in two ionic forms chromic [Cr (III)] and chromate [Cr (VI)] in the soil. Cr (VI) is more stable and soluble than Cr (III) and percolates deeply into the ground, which leads to groundwater pollution [8]. Like any other heavy metals, Cr is toxic to plants at higher concentrations and is not essential for plant growth metabolism [9]. Cr (VI) has been found to be more stable, and thus more toxic to plants [4]. Plants exposed to Cr stress, develop metabolic impairment and results in alteration of many physiological processes leading to the reduction of the germination rate, growth, chlorosis, stunting, and finally plant death [4, 9]. Some plant species such as *Nymphaea alba* L. [5], *Oryza sativa* L. [10], Clusterbean [11] are susceptible to Cr stress; whereas, some plants such as *Ocimum tenuiflorum* L. [4], *Jatropha curcas* L. [2] *Bacopa monnieri* L. [12], are quite tolerant. Plants have various defence mechanisms to endure such heavy metal stress. Genes involved in the secondary metabolic pathway like phenylalanine ammonia lyase (PAL) and polyphenol oxidase (PPO) [2, 13] initiate stress responses and help plants to adapt against abiotic stresses. Plant secondary metabolite accumulation is regarded as a responsive behaviour of plants to tolerate stress [14].

In this study, *Plantago ovata* Forsk. was chosen as a test system. It is mainly grown in India, Iraq, Canary Island and Spain [15]. In India, it is cultivated in Gujarat, and as discussed earlier, Cr pollution is quite evident in Gujarat [7]. Seed husk of *P. ovata* is widely used as a laxative to cure the intestinal disorder. It also has various other medicinal properties [16, 17]. Many studies have been conducted related to the effect of heavy metal exposure in the genus *Plantago*. Serrano et al. [18] have corroborated the heavy metal hyperaccumulation trait among *Plantago* genus. Several species of *Plantago* show hyperaccumulation of various metal pollutants like aluminium (Al), zinc (Zn), copper (Cu), lead (Pb) [18]. *Plantago arenaria* is tolerant to Cu, cadmium (Cd), nickel (Ni) and Zn [19]. Khan et al. [20] considered *P. ovata* as a hyperaccumulator of Pb, as it can grow in soil with concentration up to 4 mM. Since effects of Cr (VI) on *P. ovata* have not been studied yet; the present study was conducted to understand the underlying mechanisms of Cr (VI) toxicity towards *P. ovata* by observing the morphological and physiological responses towards the stress.

## Method

### Tissue culture media preparation

Sucrose-agar media were prepared with 3% (*w*/*v*) sucrose and 0.9% (w/v) agar. Ten seeds were transferred on to 10 ml of the prepared media contained in each 50 ml culture tubes (Borosil). Sublethal concentrations (0.1 mM, 0.3 mM, 0.5 mM, 1 mM, 1.5 mM and 1.8 mM) of potassium dichromate (SRL, India) were also added in the media. Control media was kept devoid of potassium dichromate. The culture media were heat-sterilized using autoclave.

### Seed sterilization and transfer

*Plantago ovata* seeds were imbibed overnight and then surface-sterilized for 20 min with 20% (*w*/*v*) sodium hypochlorite (NaOCl). Seeds were washed with autoclaved double-distilled water for five times (each of 5 min duration) to wash off the excess bleach. Following inoculation of the seeds in the culture media, the culture tubes were kept in a controlled environment (22 °C − 25 °C temperature, humidity of about 55% − 60%) under an artificial light intensity of 1500 lx, kept on cycle for 16 h light and 8 h dark period every 24 h. Seeds were allowed to grow for 10 days following the above growth conditions and tissue culture technique from the reported literature of Das (Pal) and Sen Raychaudhuri [21].

### Determination of root and shoot length

Ten days-old seedlings were uprooted from sucrose-agar media keeping the root intact. The root and shoot length were measured using graph paper (where, 1 cm = 1 unit). The observations were repeated three times and the mean values were represented in S.I. unit.

### Seed germination studies

*Plantago ovata* seeds were surface-sterilized with 20% (*w*/*v*) sodium hypochlorite for 20 min and then rinsed thoroughly with distilled water. The seeds were then placed for germination on filter paper-lined Petri dishes (15 cm diameter) at 25 °C under artificial light intensity of 1500 lx, kept under cycle for 16 h light and 8 h dark period (in 24 h) for two days. Each Petri dish had 10 ml of an aqueous solution of potassium dichromate, containing the specified sublethal concentrations. The data was recorded for three replicates of each treatment.

### Plant biomass calculation

Seeds were thoroughly surface-sterilized, followed by germination in sucrose-agar media (with 3% sucrose and 0.9% agar). After ten days of growth, four seedlings for each dose of potassium dichromate were gently uprooted and fresh weight (FW) was recorded. The seedlings were then oven-dried overnight at 70 °C and dry weight (DW) was recorded. The experiment was repeated three times.

### Preparation of plant tissue extracts

Shoots of *P. ovata* were weighed in a high precision balance (Wensar PGB 100) for 100 mg of fresh tissue, then crushed and homogenized with the pestle in mortar using 1 ml 50% HPLC grade ethanol (Merck, Germany). The homogenized mixture was then subjected to sonication (VC 300, Vibra Cell, Sonic materials) for 20 min followed by centrifugation at 10,000×g for 5 min. The supernatant was collected for further biochemical analysis.

### Determination of total polyphenol content

Folin-Ciocalteu reagent was used to determine the total polyphenol content spectrophotometrically, as described by Singleton et al. [22] with minor modification. Fifty μl of plant extract was mixed with 250 μl of Folin-Ciocalteu reagent and 750 μl of 10% sodium carbonate (Na_2_CO_3_), and then the mixture was shaken well and kept in the dark for 30 min. Absorbance was measured at 760 nm using JASCO V-630 spectrophotometer. The concentration of polyphenol content was determined from the gallic acid (Sigma-Aldrich, USA) calibration curve and expressed as milligram (mg) gallic acid equivalent (GAE)/ gram (g) fresh weight.

### Estimation of total antioxidant activity

The phosphomolybdenum method of Prieto et al. [23] was used to estimate total antioxidant activity of the samples. Phosphomolybdenum buffer was prepared by mixing 0.6 M sulphuric acid (Merck, Germany), 28 mM sodium phosphate (Merck, Mumbai) and 4 mM ammonium molybdate (Himedia, India) using autoclaved distilled water. Plant extract of 0.3 ml was mixed with 3 ml of the prepared buffer and then incubated at 95 °C for 90 min. The assay works on the principle that molybdenum (VI) is reduced to molybdenum (V) by the plant extract and forms a green phosphomolybdate complex [24]. Absorbance was measured at wavelength (λ_max_) of 695 nm. Total antioxidant activity was estimated as milligram ascorbic acid equivalents (AAE) per gram of the fresh weight of *P. ovata* tissue using the ascorbic acid calibration curve.

### Determination of chlorophyll and carotenoid content

The method of Sestak [25] and Lichtenthaler [26] was followed with minor modifications for total chlorophyll and carotenoids determination. Shoot tissue (100 mg) from chromium (VI)-treated seedlings was crushed and homogenized with a pestle using ice-cold acetone in a mortar. The total volume was adjusted to 5 ml with ice-cold acetone (SRL, India). The mixture was then centrifuged using a cold centrifuge machine (4 °C) for 10 min at 7728×g. The supernatant was collected and absorbance was measured according to the following equations:

$$ Chlorophyll(a)=11.24\left({A}_{662}\right)-2.04\left({A}_{645}\right) $$

$$ Chlorophyll(b)=20.13\left({A}_{645}\right)-4.19\left({A}_{662}\right) $$

$$ Total\ chlorophyll=7.15\left({A}_{663}\right)+18.71\left({A}_{646}\right) $$

$$ Carotenoids=\left[1000{A}_{470}-1.90\  Chlorophyll(a)-63.14\  Chlorophyll(b)\right]/214 $$

### Determination of DPPH radical scavenging activity

The method of Brand-Williams et al. [27] of DPPH (1, 1-diphenyl-2-picrylhydrazyl) radical scavenging assay was followed with minor modifications. The assay works on the principle of reducing red-coloured stable free radical DPPH to DPPH-H, which turns to yellow upon reduction. The antioxidants present in the plant extract have the ability to scavenge the free radicals causing change in coloration and reduction of DPPH to DPPH-H [24]. DPPH working solution (10 ml of stock solution dissolved in 45 ml methanol) was prepared from the DPPH stock solution (prepared using 24 mg DPPH dissolved in 100 ml methanol and stored at − 20 °C). Plant extract of 150 μl was mixed with 2.85 ml DPPH and incubated in the dark for an hour at room temperature. Absorbance was measured at 517 nm using a UV-VIS spectrophotometer. The ability of the plant extract to quench the free radicals was calculated by the following formulae:

$$ DPPH\ radical\ scavenging\ activity\left(\%\right)=\left[\left({A}_{control}-{A}_{sample}\right)/{A}_{control}\right]\times 100 $$

where A_control_ is the absorbance of the DPPH reagent without any sample extract and A_sample_ is the absorbance of the DPPH reagent along with each of the plant extract.

### Estimation of lipid peroxidation

An increase in the production of reactive oxygen species (ROS) due to heavy metal stress, results in its reaction with the hydrogen of the methylene groups present in the polyunsaturated fatty acid of the cell membrane, thus, causing lipid peroxidation. This phenomenon causes damage to the membrane structure and function leading to the formation of aldehyde by-product such as malondialdehyde (MDA), the thiobarbituric reactive substance (TBARS) and 4-hydroxy-2-nonenal (HNE) [28].

The MDA content was measured to determine the lipid peroxidation state due to the Cr (VI) stress on *P. ovata* seedlings. Heath and Packer’s method [29] was followed with some minor modifications. Plant tissue (*P. ovata* shoot tissue weighing 100 mg) from each sample group was crushed in 1 ml of 0.1% trichloroacetic acid (TCA) (SRL, India) followed by centrifugation of 1 ml homogenized tissue for 14 min at 300×g. One ml of the supernatant from each sample was mixed with trichloroacetic acid-thiobarbituric acid (TCA-TBA), 0.5% (*w*/*v*) TBA (SRL, India) mixed with 20% (w/v) TCA, to form TCA-TBA solution. The mixture in the test tubes was allowed to heat at 95 °C in a water bath for 30 min and then cooled immediately on ice. Absorbance was measured at 532 nm against the TCA-TBA blank and non-specific absorbance was taken at 600 nm. Lipid peroxidation was expressed as MDA equivalents as micromoles/ litre per gram fresh weight of the plant tissue. MDA extinction coefficient was taken as 155 mmol L^− 1^ cm^− 1^ [24].

### Phenylalanine ammonia lyase (PAL) activity assay

Enzyme extract was subjected to PAL activity assay using the spectrophotometric method described by Kovácik et al. [30]. *Plantago ovata* seedlings (0.3 g) were homogenised in 2 ml 0.1 M sodium borate buffer (pH 8.8) using a mortar-pestle. Homogenates were centrifuged (15000×g) at 4 °C for 15 min and the collected supernatant was used as the enzyme extract. Sodium borate buffer (500 μl) and enzyme extract (350 μl) were pre-incubated at 40 °C for 5 min. The reaction was then initiated by adding 300 μl of 50 mM l-phenylalanine. The reaction mixture was incubated at 40 °C for 60 min, and then 50 μl 5 N HCl was added to stop the reaction. Absorbance was measured at 275 nm, using the UV-Vis spectrophotometer (UV-1800, Shimadzu) to determine the amount of cinnamic acid formed. The enzyme activity was expressed as μmol trans-cinnamic acid per min per gram FW of shoot tissue. Each sample was assayed in triplicate.

### Polyphenol oxidase (PPO) activity assay

Enzyme activity was subjected to PPO assay and its activity was determined using a spectrophotometric method described by Arnnok et al. with minor modifications [31]. *Plantago ovata* seedlings (0.2 g) were ground thoroughly using a mortar-pestle in 0.1 M phosphate buffer (pH 7.0) containing polyvinylpyrrolidone. Homogenates were centrifuged at 1000×g for 20 min. The supernatant was collected and then filtered through Whatman No. 42 filter paper. The filtrate was used as enzyme extract. Fifty μl of enzyme extract, 1.95 ml of 0.1 M phosphate buffer (pH 7.0) and 1 ml of 0.1 M catechol were taken into a test tube and mixed. Absorbance was measured at 410 nm continuously for 5 min using the UV-Vis spectrophotometer (V-630, Jasco). Each sample was assayed in triplicate. The enzyme activity was expressed as an increase in absorbance units (AU) per min per gram FW of shoot tissue.

### Primer designing for sequencing of polyphenol oxidase (*PPO*) gene

Amino acid sequences of polyphenol oxidase gene of the related family of *P. ovata*, were obtained from the NCBI database and aligned. Clustal W2 software was used to find the conserved domain among the sequences. Primer3plus and Sigma DNA calculator were used to evaluate the parameters of the designed primer (Additional file 1: Table S1).

### Genomic DNA extraction and polymerase chain reaction (PCR)

Extraction of DNA was done according to Edward et al. [32] with minor modifications. The primer designed above was used to amplify our desired gene (i.e., *PPO*). Specific PCR condition was maintained as shown in Additional file 1: Table S2. After amplification, the PCR product was subjected to agarose gel electrophoresis (1.5% agar). Bio-Rad Molecular Imager (Gel Doc XR, Milan, Italy) was used to capture the gel image. The PCR product was sequenced and submitted to GenBank (Additional file 1: Table S2).

### Primer designing for expression analysis

Specific primers for expression analysis were designed with the help of Primer3plus software from the above reported sequence of *PPO* gene (GenBank accession no: KM192264.1) in our lab (Additional file 1: Table S3). Primers used for *PAL* gene expression and the endogenous control *β-Actin* [24] are also given in Additional file 1: Table S3.

### RNA extraction and reverse transcription PCR (RT-PCR) for expression analysis

The Pure link RNA mini kit (Ambion, Thermo Fisher Scientific) was used to extract RNA from *P. ovata* leaves following the instruction manual. Spectrophotometric analysis was done to check the RNA purity and integrity. Specific primers for each gene were used to run the RT-PCR to examine the gene expression using the One-step RT-PCR kit (Qiagen). Reaction conditions were maintained as given in Additional file 1: Table S4.

### Atomic absorption spectroscopy

The seedlings were uprooted from media after ten days of growth with intact roots. To avoid Cr cross-contamination from media while uprooting, the seedlings were washed with autoclaved double distilled water. The sample was then kept in a hot air oven at 70 °C overnight for drying. The dry tissue of the sample (100 mg) was digested for an overnight in a 1:1 solution of HNO_3_ and H_2_O_2_ [33]. The digested sample was then filtered through Whatman filter paper no. 41. The filtrate was allowed to evaporate by incubating at 90 °C using water bath. Then, the remaining filtrate was diluted to make a 6 ml volume with 5% HNO_3_ solution. Finally, the samples were filtered twice before measuring the absorbance at 357.8 nm in the Thermo Fisher Scientific iCE 3000 series AA spectrometer with acetylene flame. The calibration curve was prepared with chromium standard of required concentration.

### Statistical analysis

Experiments were carried out in triplicate and experimental data were expressed as mean ± SEM (standard error of the mean). The Kyplot (version 2.0) was used to obtain the statistically significant data. Experimental data were analyzed by one-way analysis of variance (ANOVA) and significance of the data was analyzed by using the Student’s t-test. A difference in experimental data at *p* ≤ 0.05 was considered statistically significant.

## Results

### Morphological analysis

**Fig. 1:**
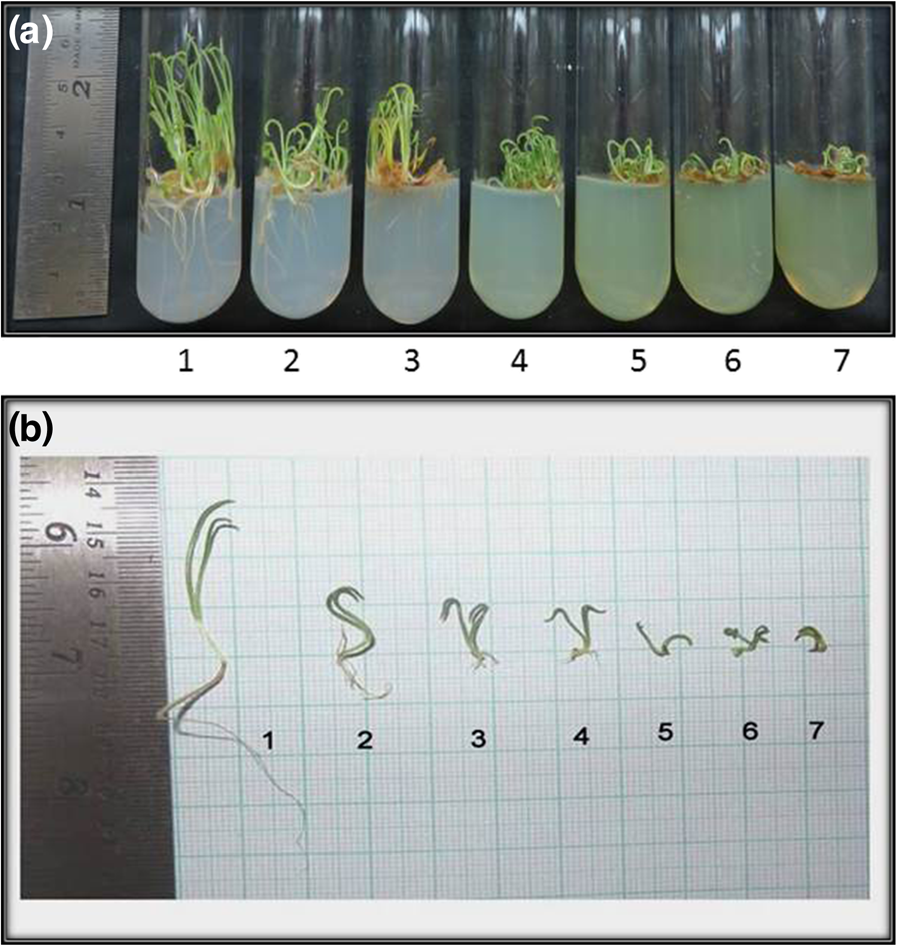
Picture of 10 day-old *P. ovata* seedlings with different doses of Cr (VI) (1) 0 mM (control), (2) 0.1 mM, (3) 0.3 mM, (4) 0.5 mM, (5) 1 mM, (6) 1.5 mM and (7) 1.8 mM; **a** *P. ovata* in agar-sucrose germination medium; **b** Measurement of shoot and root length on graph paper

**Fig 2:**
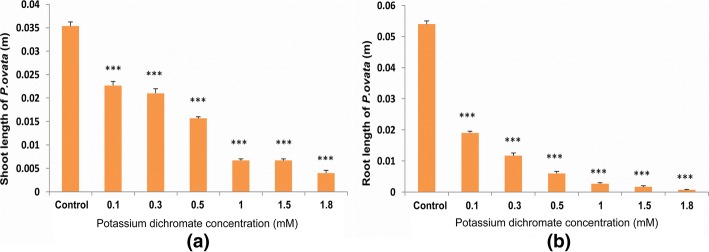
**a** Shoot length and **b** root length under Cr (VI) stress. The data are represented as mean ± standard error of mean (SEM) (*n* = 5). Asterisks denote the level of significance; *p ≤ 0.05, **p ≤ 0.01, ***p ≤ 0.001

**Fig. 3:**
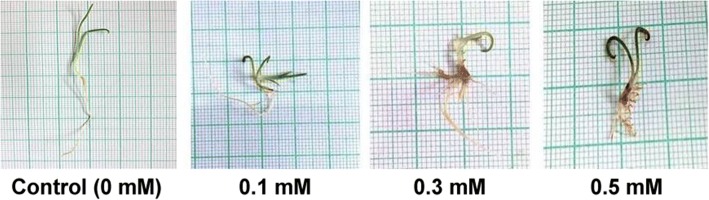
Multiple root growth in 10 day-old *P. ovata* with Cr (VI) stress (0.1 mM, 0.3 mM and 0.5 mM)

Morphological changes in the shoot and root length after exposure to Cr (VI) in the medium was quite evident (Fig. 1). The roots were more adversely affected than the shoots. At higher doses (1 mM, 1.5 mM, 1.8 mM), the shoot length was reduced below one fourth; whereas, the root length was drastically reduced below one twenty fifth compared to control (Fig. 2). Multiple root growth also occurred in the seedlings under low chromium stress (0.1 mM, 0.3 mM and 0.5 mM) (Fig. 3). Though phenotypical changes due to Cr (VI) stress affected the shoot and root length, the seedlings seemed to be quite tolerant with respect to germination rate and multiple root growth up to 0.5 mM.

### Effect on seed germination

**Fig. 4:**
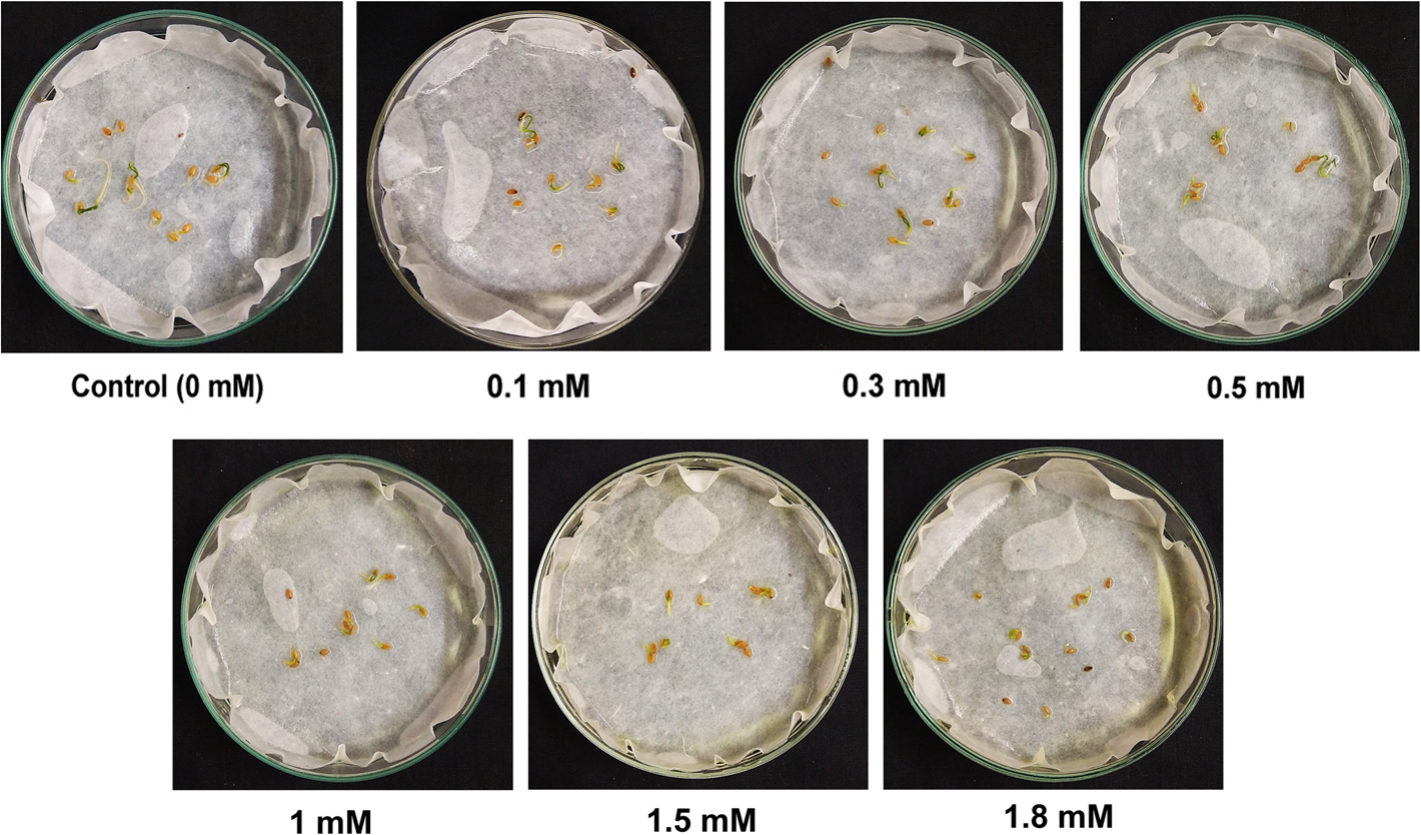
Picture of *P. ovata* seeds with different doses of Cr (VI) (1) 0 mM (control), (2) 0.1 mM, (3) 0.3 mM, (4) 0.5 mM, (5) 1 mM, (6) 1.5 mM and (7) 1.8 mM after 48 h of germination period

**Fig. 5:**
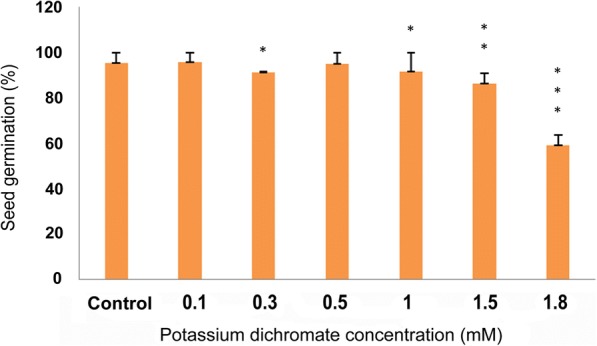
Seed germination percentage of *P. ovata* with 48 h after Cr (VI) stress. The data are represented as mean ± standard error of mean (SEM) (*n* = 5). Asterisks denote the level of significance; *p ≤ 0.05, **p ≤ 0.01, ***p ≤ 0.001

Cr (VI) had no significant effect on germination of *P. ovata* seeds at least up to 1 mM, and over this concentration, the growth of seedlings was severely suppressed (Figs. 4 and 5). After two days incubation on tissue culture media, the seed germination percentage was reduced to 86% and 59% in 1.5 mM and 1.8 mM doses, respectively, compared to 95% in seeds grown without chromium stress.

### Effect on plant biomass

**Fig. 6:**
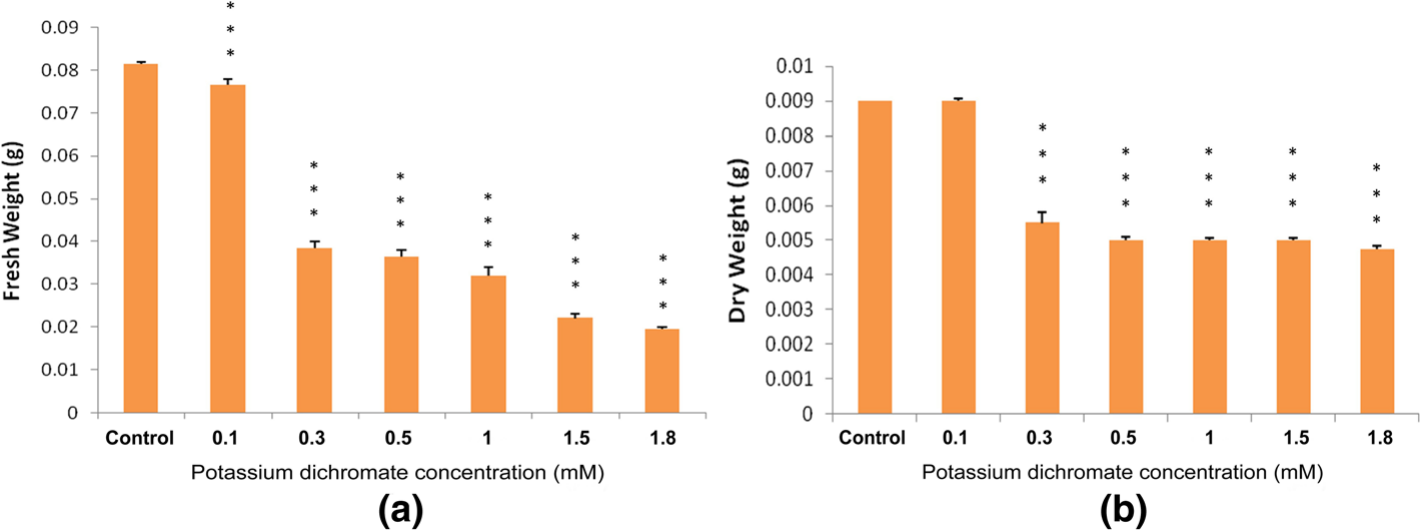
**a** Fresh weight and **b** dry weight of *P. ovata* with Cr (VI) stress after 10 days of growth. The data are represented as mean ± standard error of mean (SEM) (*n* = 5). Asterisks denote the level of significance; *p ≤ 0.05, **p ≤ 0.01, ***p ≤ 0.001

**Fig. 7:**
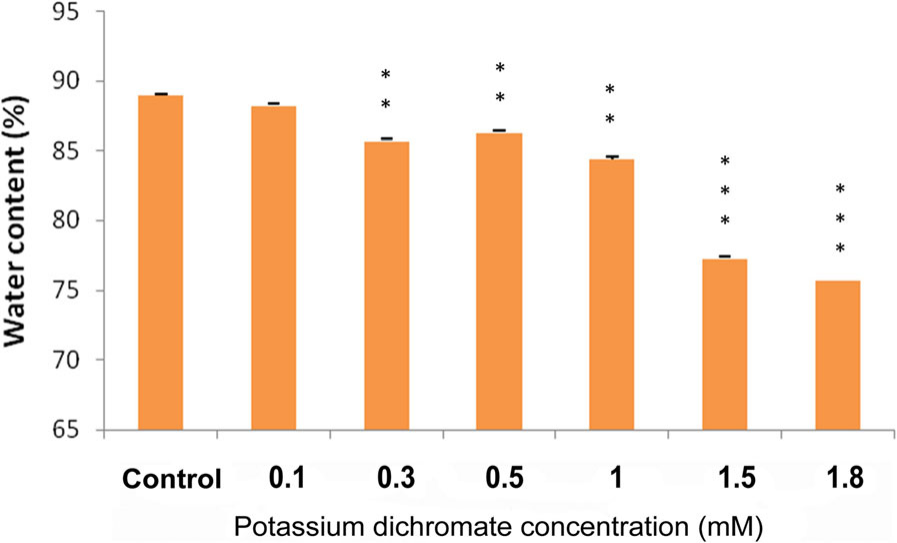
Water content (%) in *P. ovata* with Cr (VI) stress after 10 days of growth. The data are represented as mean ± standard error of mean (SEM) (*n* = 5). Asterisks denote the level of significance; *p ≤ 0.05, **p ≤ 0.01, ***p ≤ 0.001

Cr (VI) caused a negative impact on *P. ovata* biomass production (Fig. 6). The effect was drastic at 0.3 mM dose, where fresh weight (FW) reduced by a half compared to the control seedlings. At the Cr (VI) concentrations higher than 0.3 mM, the FW gradually decreased. The dry weight (DW) also followed the same trend and was reduced to about 63% of the control. Percentage of water content, calculated from the FW and DW revealed that the water content decreased with the increase in the Cr (VI) concentrations (Fig. 7). Plants grown at the higher concentrations (1.5 mM and 1.8 mM) severely affected the water content in the tissue.

### Chlorophyll, carotenoid and total polyphenol contents

**Fig. 8:**
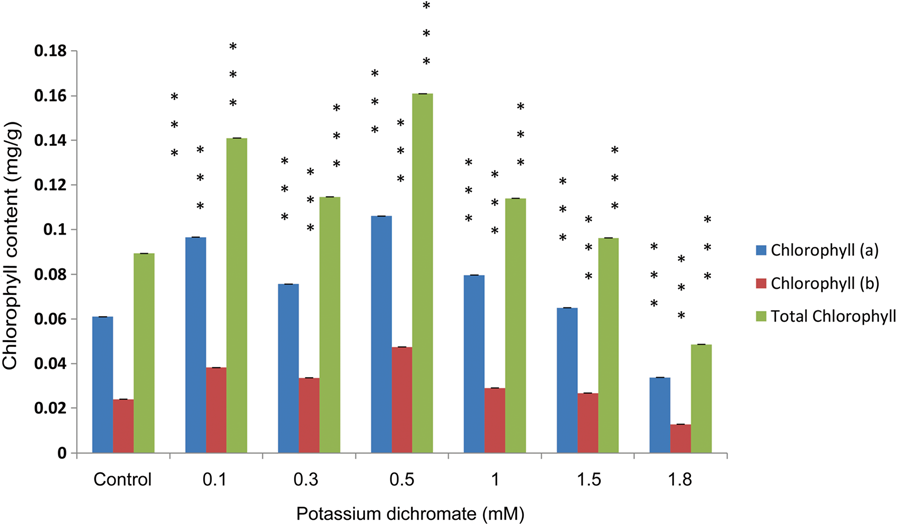
Chlorophyll a, b and total chlorophyll content in 10 day-old *P. ovata* with Cr (VI) stress. The data are represented as mean ± standard error of mean (SEM) (*n* = 5). Asterisks denote the level of significance; *p ≤ 0.05, **p ≤ 0.01, ***p ≤ 0.001

**Fig. 9:**
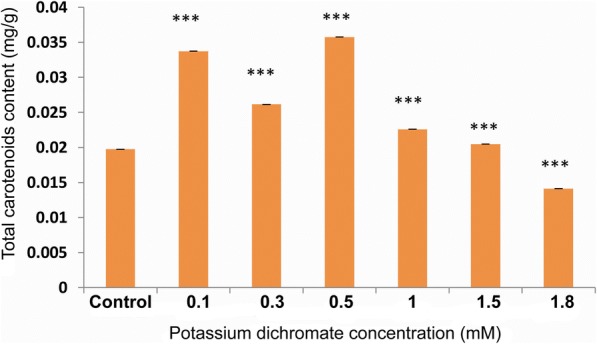
Total carotenoid content in 10 day-old *P. ovata* under Cr (VI) stress. The data are represented as mean ± standard error of mean (SEM) (*n* = 5). Asterisks denote the level of significance; *p ≤ 0.05, **p ≤ 0.01, ***p ≤ 0.001

**Fig. 10:**
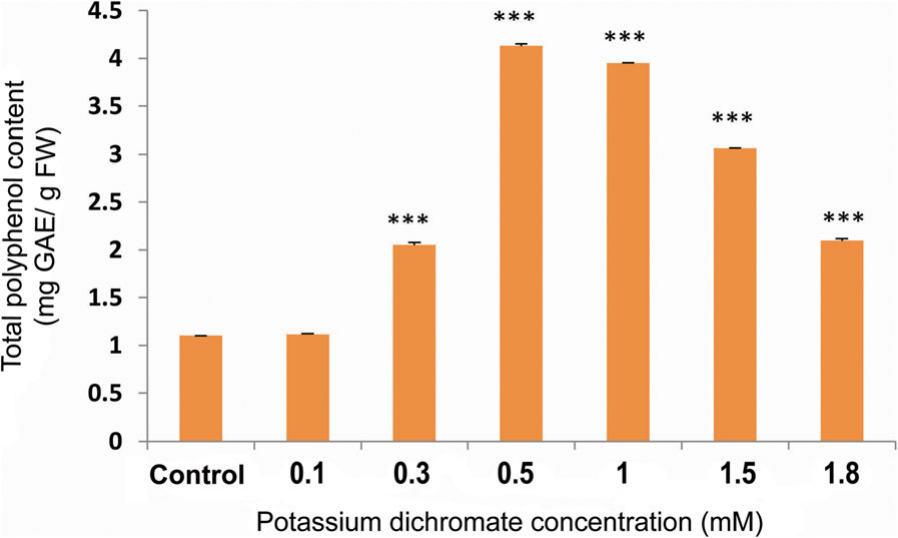
Total polyphenol content in 10 day-old *P. ovata* under Cr (VI) stress. The data are represented as mean ± standard error of mean (SEM) (*n* = 5). Asterisks denote the level of significance; *p ≤ 0.05, **p ≤ 0.01, ***p ≤ 0.001

Chlorophyll (a, b and total chlorophyll) and carotenoid contents in the seedlings increased significantly with Cr (VI) exposure up to 1.5 mM concentration. The highest and lowest contents were found at 0.5 mM and 1.8 mM Cr (VI), respectively (Fig. 8). The carotenoid content also followed the same trend as the chlorophyll content (Fig. 9). At the concentrations above 0.5 mM, there was a steady decrease in the chlorophyll as well as carotenoid contents. The total polyphenol content increased approximately 3.8 times in the seedlings treated with 0.5 mM Cr (VI) compared to the control. The total polyphenol content was highest at 0.5 mM Cr (VI) (Fig. 10).

### Total antioxidant levels and DPPH radical scavenging activity

**Fig. 11:**
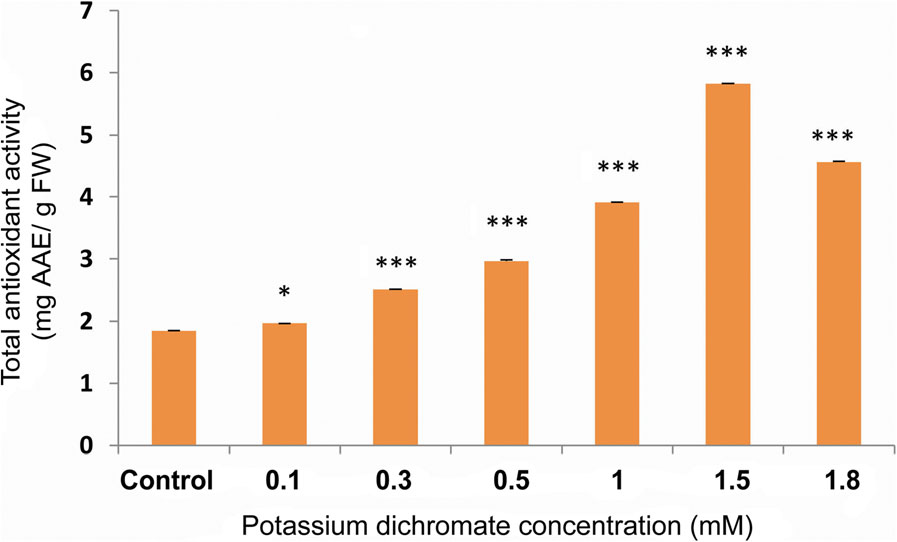
Total antioxidant activity in 10 day-old *P. ovata* with Cr (VI) stress. The data are represented as mean ± standard error of mean (SEM) (*n* = 5). Asterisks denote the level of significance; *p ≤ 0.05, **p ≤ 0.01, ***p ≤ 0.001

**Fig. 12:**
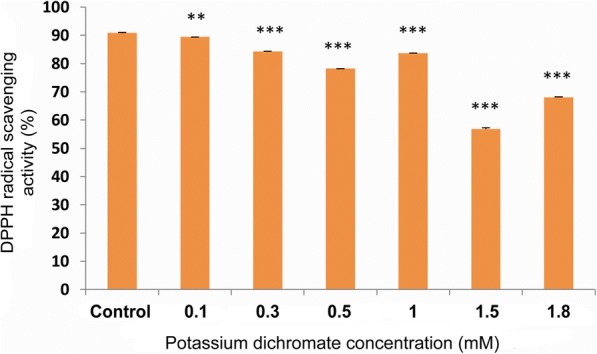
Percentage of DPPH radical scavenging activity in 10 day-old *P. ovata* with Cr (VI) stress, where, 100% DPPH radical scavenging activity refers to inhibition of all the free radicals by the sample. The data are represented as mean ± standard error of mean (SEM) (*n* = 5). Asterisks denote the level of significance; *p ≤ 0.05, **p ≤ 0.01, ***p ≤ 0.001

Steady and significant increase in total antioxidant activity up to 3 times in 1.5 mM, as compared to the control, and then slightly declined at the highest Cr (VI) concentration (1.8 mM) in the experiment (Fig. 11). Though total antioxidant content increased with the stress, DPPH radical scavenging activity, i.e., the percentage of free radical inhibition, did not increase as compared to the control. The reduction was significant, but very little up to 1 mM of Cr (VI) dose (Fig. 12). Percentage of inhibition was 90.8% in control plant followed by 89.4, 84.3, 78.2 and 83.7% with Cr (VI) dose of 0.1 mM, 0.3 mM, 0.5 mM, and 1 mM, respectively. At the concentrations 1.5 mM and 1.8 mM, the inhibition decreased sharply to 56.8% and 68.1% respectively.

### Lipid peroxidation levels

**Fig. 13:**
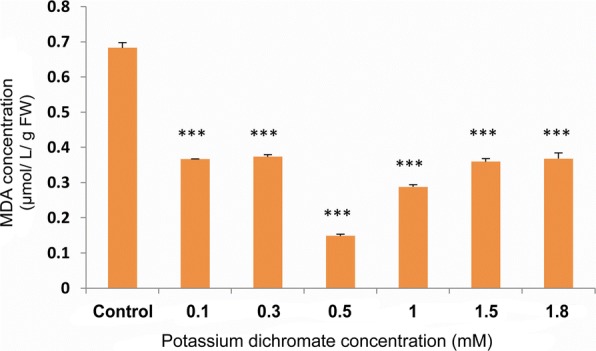
Lipid peroxidation levels with Cr (VI) stress exposure in 10 day-old *P. ovata*. The data are represented as mean ± standard error of mean (SEM) (*n* = 5). Asterisks denote the level of significance; *p ≤ 0.05, **p ≤ 0.01, ***p ≤ 0.001

The MDA content indicating the lipid peroxidation level, was significantly low in the seedlings exposed to Cr (VI) stress. The peroxidation level decreased to one half in all the Cr (VI) treated seedlings (0.1 to 1.8 mM), as compared to the control seedlings (Fig. 13).

### PAL and PPO activities

**Fig. 14:**
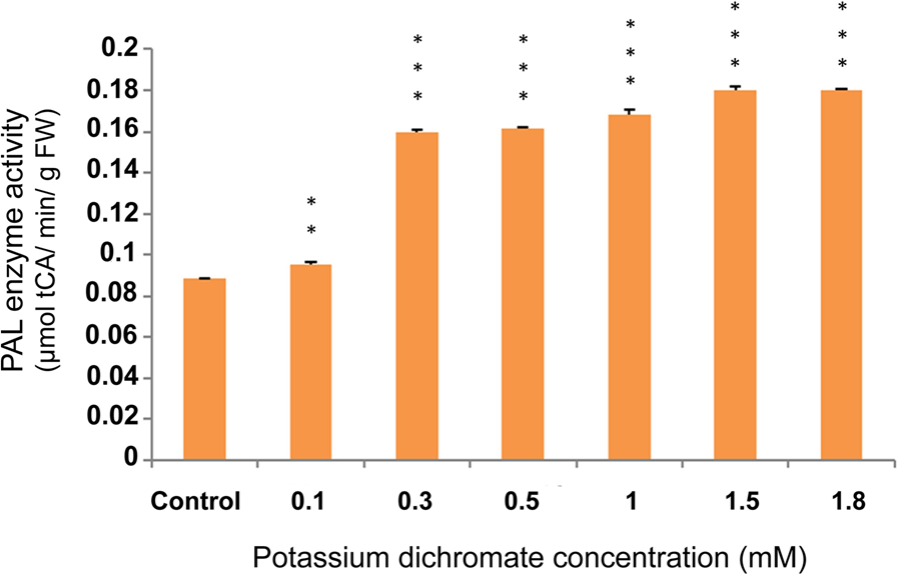
PAL activity assay with Cr (VI) stress exposure in 10 day-old *P. ovata*. The data are represented as mean ± standard error of mean (SEM) (*n* = 5). Asterisks denote the level of significance; *p ≤ 0.05, **p ≤ 0.01, ***p ≤ 0.001

PAL activity assay was carried out to measure the changes in the concentration of PAL enzyme in the seedlings treated with various Cr (VI) concentrations, and the result was expressed as μmol trans-cinnamic acid min^− 1^ g^− 1^fresh weight (FW) of callus. PAL activity increased gradually from 0.3 mM that is 1.8 times higher than the control level, and saturated at 1.5 mM and above (Fig. 14).

**Fig. 15:**
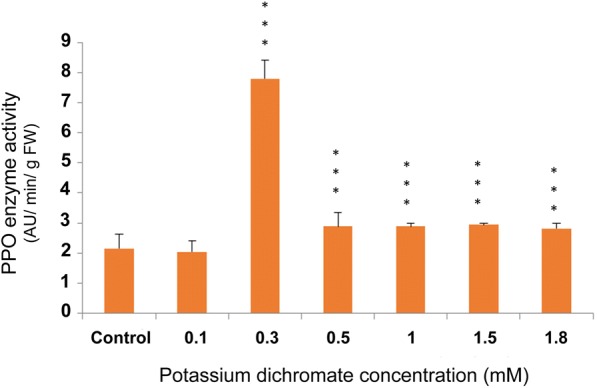
PPO activity assay with Cr (VI) stress exposure in 10 day-old *P. ovata*. The data are represented as mean ± standard error of mean (SEM) (*n* = 5). Asterisks denote the level of significance; *p ≤ 0.05, **p ≤ 0.01, ***p ≤ 0.001

PPO activity assay was carried out to measure the amount of PPO enzyme in the samples at the various Cr (VI) concentrations, and the result was expressed as absorbance units (AU) min^− 1^ g^− 1^ fresh weight of shoot tissue. The PPO activity became maximum at 0.3 mM that is 3.6 times higher than the activity of the control (Fig. 15), and then the activity gradually dropped down to the control levels till 1.8 mM.

### *PAL* and *PPO* gene expressions

**Fig. 16:**
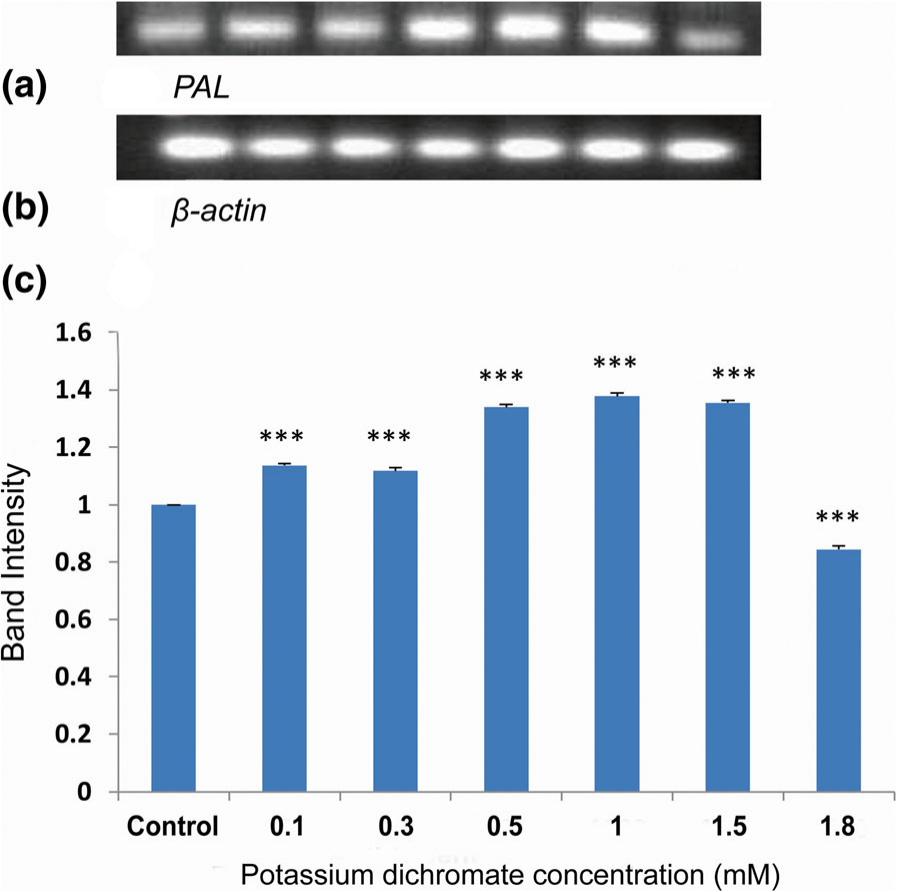
Gel picture of (**a**) *PAL* gene expression; (**b**) *β-actin* expression as endogenous control; (**c**) Densitometry of the *PAL* gene expression

Phenylalanine ammonia lyase (*PAL*) gene expression was significantly upregulated in the seedlings treated with Cr (VI). RT-PCR band intensity was enhanced with increasing Cr (VI) concentrations. The *PAL* gene expression was highest at 1 mM Cr (VI) of which intensity was 1.4-fold compared to control (Fig. 16).

**Fig. 17:**
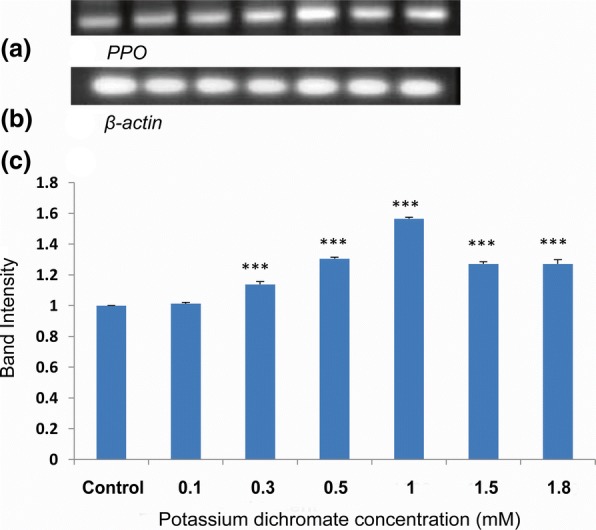
Gel picture of (**a**) *PPO* gene expression; **b** *β-actin* expression as the endogenous control; **c** Densitometry of the *PPO* gene expression

Expression of polyphenol oxidase (*PPO*) gene, an anti-oxidative enzyme gene responsive to the heavy metals, was also upregulated with Cr (VI) treatment (Fig. 17) [2, 11]. The upregulation was highest at 1 mM Cr (VI) of which expression was 1.6-fold compared with the control.

### Chromium accumulation

**Fig. 18:**
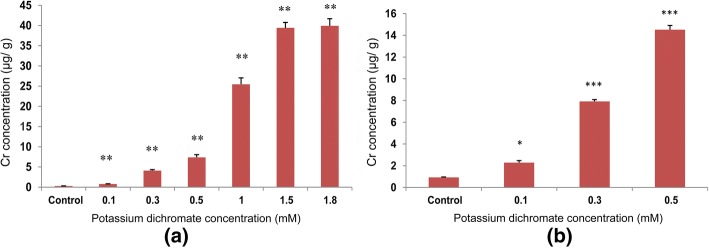
Chromium accumulations in 10 day-old *P. ovata* (**a**) shoot and (**b**) root, measured by AAS. The data are represented as mean ± standard error of mean (SEM) (*n* = 5). Asterisks denote the level of significance; *p ≤ 0.05, **p ≤ 0.01, ***p ≤ 0.001

Chromium accumulation in shoot and root of *P. ovata* shoots and roots was enhanced with increasing Cr (VI) concentration in the germination medium (Fig. 18). Control shoot tissues devoid of Cr (VI) in the medium contained 0.27 ppm Cr (VI), while 0.1 mM (29.4 mg/L) Cr (VI) treated seedlings, showed increased Cr accumulation by 2.9 times in the shoot. The accumulation of Cr (VI) increased by 147.6 times of the control. In the control root tissues, the Cr (VI) content was 0.93 ppm that was higher than the Cr (VI) content found in the control shoots. Accumulation of Cr (VI) in the root also increased in a dose-dependent manner with increasing Cr (VI) concentrations in the culture medium. With 0.5 mM Cr (VI) treatment, its accumulation in the roots increased by 15.6 times of the control.

## Discussion

Heavy metal stress induces an alteration in many physiological processes and affects many biochemical pathways resulting in decrease or increase in production of various metabolic compounds. Like other heavy metals, potassium dichromate (Cr VI) treatment resulted in significant reduction of the shoot and root length in 10 day-old *P. ovata* seedlings in a dose-dependent manner (Fig.1). High doses of Cr (VI) stress mainly affected the radical growth in *P. ovata*, similar to the findings by other investigators [1, 34]. Root growth was mainly affected through tissue damage which hindered water and nutrient absorption from the media containing Cr (VI) [1, 35]. In spite of reduction in root length, it should be noted that multiple root development was found in *P. ovata* with 0.1–0.5 mM Cr (VI) concentrations (Fig. 3), which have been found previously in Cr (VI)-treated alfalfa plants [1, 36]. Root growth affects biomass, length, amount and rate of elongation of plants depending on the tolerable or toxic levels of heavy metals in the soil [37]. In *P. ovata*, the shoot length and root length decreased with increasing Cr (VI) concentrations (Fig. 2), which would be due to the inhibition of cell division possibly by inducing chromosomal aberrations, as observed by Liu et al. [38]. Seed germination was not affected significantly at the low concentrations of Cr (VI) (Figs. 4 and 5). Increase in various factors such as α-amylase, an enzyme involved in seed germination to supply sugar, may block the adverse effects of low doses of Cr (VI) on the seed germination [39]. The rate was affected by only above 1 mM, wherein it decreased by 1.5 times at the highest dose (1.8 mM).

The fresh weight of the plant tissue was severely affected by the treatment with Cr (VI), which reduced severely even at lower concentrations (Fig. 6). Our results are consistent with the results of Wyszkowski et al. [40], which shows that the biomass of spring barley decreased; with an increase in the Cr content. These results might imply that the reduction in the content of the vital pigments inhibits the growth of the plant. Water content also decreased (Fig. 7), which is quite evident from the fact that the roots were severely affected by Cr (VI), and thus, they impaired further water absorption and transport from the culture media.

Heavy metal stress affects various growth parameters like, inhibiting physiological processes by hampering pigment formation and degrading chloroplast ultrastructure. Chromate has been known to influence the enzymes of the Calvin cycle and was even used as a Hill reagent [9, 41, 42]. Here in *P. ovata*, chlorosis was not observed at the low concentrations of Cr (VI), thus, showing a significant increase in total chlorophyll formation, i.e., 1.8 times with 0.5 mM Cr (VI) to that of the control seedlings (Figs. 8 and 9). Subsequently in the present investigation, chlorophyll a, b and carotenoid contents increased in the seedling tissue. The results are quite different from majority of other documented findings that showed that even lower doses of Cr stress affected the pigment formation and formed chlorosis like in *Nymphaea alba* L. [5] and *Oryza sativa* L. [10]. But leaves and stems of some plants like *Jatropha curcas*, showed elevated chlorophyll a content along with chlorophyll b, total chlorophyll, and carotenoid under 50 and 100 mg/kg Cr stress. Similarly, *Plantago arenaria* did not show any sign of chlorosis or wilting under the heavy metal stress like Cu, Zn, Cd, and Ni and is found to have tolerance against heavy metals stress [19]. *P. ovata* seedlings also showed such adaptive response via pigment restoration and formation, up to certain level of Cr (VI) concentration, implying the increase in the chlorophyll content as a plant defence responses against the stress.

Total polyphenol content was also enhanced by 3.8 times in *P. ovata* seedlings when treated with 0.5 mM Cr (VI) concentration, with respect to the untreated set (Fig. 10). Polyphenols are a group of plant secondary metabolites which are known to be accumulated as a part of stress response, thus, making the plant capable of withstanding any harsh condition [12, 43]. In the varieties of *Catharanthus roseus*, secondary metabolites like vincristine and vinblastine were enhanced by Cr stress [44], but the effect of Cr on polyphenol content has not been investigated much. Increase in the polyphenol content under other heavy metal stresses was quite evident in various studies. In *Jatropha curcas*, Pb and Cr treatment enhanced the phenolic content in all parts of the plant [2]. Also, in a recent study, Cd treatment on *Gynura procumbens* resulted in enhanced phenolic accumulation [45]. Moderate Cd dose affected the growth parameters in *H. hemerocallidea* along with an increase in the polyphenol accumulations, though low Al levels did not have any affect [43]. These results including our data suggest that polyphenol accumulation is the outcome of the plant’s robust mechanism to fight and adapt against the metal stress.

Total antioxidant activity of *P. ovata* was enhanced by Cr (VI) stress by 3.2 times with 1.5 mM (Fig. 11). In camomile plants, excess Cr treatment caused increase in antioxidant enzyme and ascorbic acid content [46]. In *Sorghum bicolour* L., Al treatment caused increase in antioxidants like glutathione (GSH) [47]. Therefore, it can be inferred that increased total antioxidant levels as well as total polyphenol content depict an adaptive behaviour of *P. ovata* against Cr (VI) stress.

There was a significant decrease in total free radical scavenging activity with increasing concentration of Cr (VI) stress in *P. ovata* (Fig. 12) due to generation of free radicals. Plants generate various secondary metabolites that can act as antioxidants [48], but the synergistic effects of these antioxidants are not always enough to quench the free radical pool. Thus, plants are trying to combat the stress to some extent but not completely, resulting in morphological alterations like reduction in shoot and root length. Lipid peroxidation of cell membrane is also free radical damage generated by the heavy metal stress. MDA forms as an oxidized product of the polyunsaturated fatty acid present in the membranes of cells, and so, its content acts as an indicator of the oxidative damage [29, 49]. The MDA content was found to be low in Cr (VI) treated seedlings when compared to the control group, which is quite similar to the findings that lipid peroxidation levels of *Cajanus cajan* L. decrease with an increase in antioxidant enzyme activity [50]. These results might suggest that the membrane damage by free radical generation was not prominent possibly due to the increase in the total antioxidant levels in *P. ovata* (Fig. 13).

*Polyphenol oxidase* (*PPO*) and *Phenylalanine ammonia lyase* (*PAL*) gene expressions along with their enzyme activity elevated with an increase in Cr (VI) concentration in *P. ovata* seedlings. There was a 1.6-fold increase in *PPO* gene expression with 1 mM Cr (VI), which is the highest increase among other concentrations (Fig. 17). PPO enzyme activity also followed the similar trend with its gene expression (Fig. 15). Polyphenol oxidase has a role in plant defence response against various abiotic stresses [51], and so, the upregulation in the expression of the *PPO* gene expression may have played an important role in *P. ovata* adaptive response against Cr (VI) stress. Similar results were found in *Jatropha curcus* L., where polyphenol oxidase activity was elevated by Cr (VI) treatment [2]. PAL is one of the key enzymes with a vital role in the phenylpropanoid pathway, and catalyzes the initial step in the synthesis of various plant secondary metabolites. So, change in the *PAL* gene expression due to heavy metal stress was already corroborated in various literatures. In our study, elevation of *PAL* gene expression and its enzyme activity by Cr (VI) stress was found in *P. ovata* (Figs. 14 & 16). In other studies, the elevation of *PAL* activity by Cr (VI) and Al (III) stresses was found in *Jatropha curcus* L. and *Sorghum bicolor*, respectively [2, 47].

In this study, *P. ovata* was found to be more or less tolerant to chromium (VI) stress, which was evident from the increase in plant secondary metabolites [52], total antioxidant activity, reduction in lipid peroxidation along with upregulation in *PPO* and *PAL* gene expressions as well as its enzyme activity under chromium (VI) application. These results support the fact that, the plant tries to quench the ROS generation using secondary metabolite production to combat the stress. The quantity of Cr uptake in *P. ovata* was not comparable to other Cr hyperaccumulators (Fig. 18). At 1 mM Cr (VI), *P. ovata* accumulated 25.4 ppm (or 25.4 mg/kg) Cr in the shoot, which is about 8.3% of the total metal concentration given in the medium. At 1.8 mM Cr dose, the uptake was 40 ppm (or 40 mg/kg). This result may depict similarity in Cr accumulation capacity of other *Plantago* species, *P. algarbiensis*, *P. almogravensis, P. holosteum, P. alpina, P. maritime, P. coronopus, P. serraria, P. asiatica, P. major, P. australis, P. afra, P. arenaria, P. bellardii, P. lagopus and P. lanceolata* [18]. The magnitude of Cr (VI) uptake of Plantago would be poor compared to *Leersia hexandra* Swartz, a hyperaccumulator of Cr [53].

Though Cr pollution is pertinent in soils of Gujarat near industrial area, *P. ovata* grows in agricultural fields, where Cr contamination is low. This study gave us information of the plant’s behaviour in the presence of Cr (VI) stress and provided us with an outlook of measures to take care of the polluted soil before cultivating *P. ovata*.

## Conclusion

The present study on *P. ovata* under the presence of Cr (VI) gave us important insights about how the plant system deals with the stress. Increase in plant secondary metabolites, total antioxidant levels, upregulation of *PAL* and *PPO* genes as well as the enzymatic activity in the plant depict the survival strategy and response towards the increase in Cr (VI) stress*,* thus reinforcing that these metabolites and genes play to combat the stress. This study will help us to elucidate the target secondary metabolic pathway for the plant defence response against such the heavy metal stress.

## Additional file

Additional file 1: **Table S1.** Designed primers for sequencing of *PPO* gene. **Table S2.** PCR conditions of *PPO* gene amplification for sequencing. **Table S3.** Primers for expression analysis. **Table S4.** Reaction conditions for expression analysis. (DOCX 15 kb)

## Abbreviations

Cr (VI): Hexavalent chromium
Cr: Chromium
DPPH: 1, 1-diphenyl-2-picryl-hydrazyl
MDA: Malondialdehyde
PAL: Phenylalanine ammonia lyase
PPO: Polyphenol oxidase
TBARS: Thiobarbituric reactive substance
