# Supplementary material for: Chromium (VI) – induced stress response in the plant Plantago ovata Forsk in vitro
Source: Genes Environ. 2018 Oct 15;40:21. doi: 10.1186/s41021-018-0109-0 (PMC6192006; doi:10.1186/s41021-018-0109-0)
Supplement: Supplementary file 1 — Table S1. Designed primers for sequencing of PPO gene. Table S2. PCR conditions of PPO gene amplification for sequencing. Table S3. Primers for expression analysis. Table S4. Reaction conditions for expression analysis. (DOCX 15 kb) [file 41021_2018_109_MOESM1_ESM.docx]

**Table S1** Designed primers for sequencing of *PPO* gene.

| **Gene** | **Forward Primer (5’- 3’)** | **Reverse Primer (5’- 3’)** |
| --- | --- | --- |
| *PPO* | GGCTCTTCTTCCCCTTCCA | ACGAGCGGCGGAGTAGAA |

**Table S2** PCR conditions of *PPO* gene amplification for sequencing.

| **Gene** | **Denaturation** | **Annealing** | **Elongation** | **Final extension** | **No. of cycle** |
| --- | --- | --- | --- | --- | --- |
| *PPO* | 94 °C  for  1 min | 53 °C  for  1 min | 72 °C  for  1min 30 sec | 72 °C  for  10 min | 35 |

**Table S3** Primers for expression analysis

| **Gene** | **Forward Primer (5’- 3’)** | **Reverse Primer (5’- 3’)** |
| --- | --- | --- |
| *PPO* | CCCAAAAGCCAGTCCTCCAT | AGAACCACGCTCGGGATGAC |
| *PAL* | AAGAACGGCGAACATGAGAAG | GATCCGATTCGCGATTGCT |
| *β-actin* | ATCATGAAGTGTGATGTTGA | ACCTTAATCTTCATGCTGCC |

**Table S4** Reaction conditions for expression analysis

| **Gene** | **Reverse Transcription** | **Initial Denaturation** | **Denaturation** | **Annealing** | **Elongation** | **Final extension** | **No. of cycle** |
| --- | --- | --- | --- | --- | --- | --- | --- |
| *PPO* | 50 °C,  30 min | 95 °C,  15 min | 94 °C,  1 min | 60 °C,  1 min | 72 °C,  1 min | 72 °C,  10 min | 40 |
| *PAL* | 50 °C,  30 min | 95 °C,  15 min | 94 °C,  1 min | 60 °C,  1 min | 72 °C,  1 min | 72 °C,  10 min | 40 |
| *β-actin* | 50 °C,  30 min | 95 °C,  15 min | 94 °C,  1 min | 60 °C,  1 min | 72 °C,  1 min | 72 °C,  10 min | 40 |
